# Diet Quality and Physical Activity and Their Association with BMI and Dental Caries Among High School Adolescents: A Cross-Sectional Study

**DOI:** 10.3390/children11111282

**Published:** 2024-10-24

**Authors:** Deema J. Farsi

**Affiliations:** Department of Pediatric Dentistry, Faculty of Dentistry, King Abdulaziz University, P.O. Box 80200, Jeddah 21589, Saudi Arabia; dfarsi@kau.edu.sa; Tel.: +966-12-640-2000 (ext. 20388)

**Keywords:** dental caries, BMI, obesity, adolescence, physical activity, diet, KIDMED

## Abstract

**Objectives:** The aim of this study was to assess diet quality and its association with obesity and dental caries. It also assessed adolescents’ physical activity (PA) level and its relationship with obesity. **Methods:** This cross-sectional study examined 300 high school adolescents. Body mass indices (BMI) were calculated after taking participants’ heights and weights. Caries activity was recorded as decayed, missed, and filled scores (DMFT). Diet quality was assessed using the KIDMED questionnaire, and four additional questions were added to assess junk food consumption. PA was assessed using PAQ-A. KIDMED, junk food, and PA scores were calculated. Statistical analyses included Kruskal–Wallis and Wilcoxon Rank Sum tests for group comparisons. A Generalized Linear Model (GLM) was utilized to assess predictors of BMI percentile. **Results:** The KIDMED score was 3.0 ± 2.5, with 57.7% of participants classified as having very poor diet quality and 39% categorized as needing improvement. The majority of the participants had low PA levels (81.3%), with only 18% reporting moderate PA. No associations were observed between KIDMED scores, junk food consumption with BMI percentile, DMFT, or number of decayed teeth. Children with “very poor” KIDMED scores had a BMI percentile of 58.7 ± 33.9, while those with “needs improvement” and “optimal” diets had percentiles of 60.0 ± 30.0 and 65.1 ± 35.7, respectively. Higher PA levels were associated with better diet quality (*p*-value <0.001). The regression analysis, controlling for age, sex, DMFT, KIDMED, junk food consumption, and PA score did not identify any predictors of BMI percentile. **Conclusions:** Participants consumed poor quality diets and engaged in minimal PA. No associations could be concluded between diet and PA with either BMI or dental caries. Further research is needed to better understand these relationships.

## 1. Introduction

Adolescence is the period between childhood and adulthood, which is usually between the ages of 10 and 19 years. Adolescents experience rapid growth and development, including physical, cognitive, social, and psychosocial changes [1,2]. Adolescence is not only an important time for fostering healthy habits but also a time when many behavioral and health problems begin, which may have consequences that extend into adulthood [3].

Obesity in adolescence is a serious health problem and its prevalence is increasing with time worldwide. The prevalence of morbid obesity is increasing among adolescents in the United States and Australia [4,5]. In Saudi Arabia, adolescent obesity has increased from 12.6% in 2008 to 15.3% in 2013 [6]. In the city of Jeddah, a recent study found the prevalence of overweight/obese adolescents to be 27.8% [7]. Obese children are likely to remain obese during adolescence and around 80% of obese adolescents will continue to be obese in adulthood [8]. Obesity in adolescence is a precursor to other complex diseases in adulthood, such as metabolic and cardiovascular disease, and is associated with an increased risk of several malignancies such as breast and colorectal cancer [9]. Therefore, the prevention and reduction of obesity in adolescents is of paramount importance.

Obesity is multifactorial and can be influenced by genetic, metabolic, endocrinal, dietary, behavioral, and environmental factors [10,11]. Factors leading to obesity that are specifically unique to adolescents include easy access to fast food, independence in making dietary choices, engagement in video games and screen time, failure to meet required physical activity (PA) levels, television watching, and insufficient sleep [12,13,14,15,16,17].

The World Health Organization (WHO) recommends that adolescents engage in moderate- to vigorous-intensity exercise for at least 60 min per day and incorporate vigorous-intensity activities and strengthening exercises at least three times per week [18]. In a recent meta-analysis, environmental factors that encouraged PA and discouraged sedentary behaviors were negatively associated with childhood obesity [19]. Several techniques with different applications have been used to assess PA levels in children and adolescents. These techniques include direct observations, self-reporting (questionnaires and activity logs), electronic monitoring (heart rate monitoring, accelerometers, and pedometers), and calorimetry [20]. A recent study analyzed data from global PA surveillance initiatives and identified insufficient levels of PA in children and adolescents worldwide, as well as a decline in PA levels with age [21].

Adolescents’ autonomy in choosing what and when to eat, coupled with frequent social eating and negative peer influence, has led to a marked increase in the consumption of carbonated beverages, fast foods, and nutritionally poor snacks [14,22]. Adolescents in Saudi Arabia have been found to have poor dietary behaviors and high ingestions of processed unhealthy foods that very often contain a dangerous amount of added sugar [23,24]. It is not surprising that the national prevalence of dental caries among adolescents in their permanent dentition is 61.7%, with a mean DMFT of 2.42 ± 2.52 [25].

There are about 4.74 million people between the ages of 10 and 19 in Saudi Arabia, constituting 14.6% of the total population [26]. Programs designed to prevent youth health problems would be most effective after thoroughly assessing their lifestyles and the specific factors that contribute to their vulnerability. This study aimed to assess diet quality in an adolescent population and investigate its association with obesity and dental caries. It also assessed adolescents’ PA level and its relationship with obesity.

## 2. Materials and Methods

This was a cross-sectional study and the second part of a two-part study. The target population comprised twelfth-grade senior students. Of 44,000 senior students in the city of Jeddah, Saudi Arabia (51.1% male and 48.9% female), 300 adolescents were recruited (50% male and 50% female) via convenience sampling from three private schools.

Permission to collect data was obtained from the Saudi Ministry of Education. During the first school visit, the principals were briefed about the study, and parental consent forms with a concise demographic questionnaire were distributed to all senior students. During the following school visit, adolescents who brought back a signed consent form were enrolled in the study. Adolescents with complex medical conditions and those younger than 16 years and older than 20 years were excluded. Two adolescents refused to participate and were also excluded.

The Ethics Committee of the Faculty of Dentistry at King Abdulaziz University (KAUFD) approved the study protocol (064-06-21). The data were collected between September 2022 and March 2023.

### 2.1. Anthropometric Measurements

Two trained and calibrated general dentists performed anthropometric measurements for all enrolled adolescents. Training and calibration were performed at the KAUFD with a sample of 15 adolescent patients. Kappa was 0.83 and 0.86 for inter-examiner and intra-examiner reliability, respectively.

Height was measured using a non-elastic measuring tape, with each student standing barefoot and looking straight ahead. The height was rounded to the nearest 0.1 cm. Weight was measured using a single digital scale, with adolescents standing barefoot and without heavy clothing. This was rounded to the nearest 100 g. Body mass index (BMI) was calculated using the following formula: weight in kg/height in m^2^. Saudi sex-specific and age-specific BMI percentiles with standard international cutoff points were used to categorize BMI [27,28]. Two readings for each measurement were performed and the median of these readings was used.

### 2.2. Dental Examination

Caries in the permanent teeth was recorded using the decayed, missed, and filled (DMFT) scoring criteria established by the WHO [29]. Two calibrated general dentists performed the oral examinations using disposable sterile mouth mirrors and dental probes. Dental findings were shared with the participants, and a report on the findings with contact information from KAUFD’s pediatric dentistry clinics was sent to the parents.

### 2.3. The Questionnaire

A structured questionnaire consisting of two sections was compiled from previous validated questionnaires as explained below. Its development and validation is discussed in detail in the first part of the study [30]. The questionnaire was pilot-tested on 15 adolescents aged 17 years who were not part of the study. The reliability was tested by retesting ten of them two weeks later (Kappa 0.89).

#### 2.3.1. Section I: Diet Evaluation

The updated KIDMED questionnaire was used to assess diet quality along with the original scoring system [31]. In addition, four questions that investigated the consumption of potato/corn chips, juice, flavored milk, and sugar-sweetened carbonated beverages were added; however, their analysis was performed separately from the total KIDMED score.

#### 2.3.2. Section II: Physical Activity

The Physical Activity Questionnaire for Adolescents (PAQ-A) is considered appropriate for assessing the physical activity of high school adolescents [32]. It was translated into Arabic with a minor modification in the first question (the item pertaining to sports and activities not commonly practiced in Saudi Arabia, such as skating and skateboarding, were deleted). The original PAQ-A was used in this study.

The participants completed the comprehensive 2-page questionnaire independently after their anthropometric measurements were taken. The questionnaire took approximately five minutes to complete and none of the participant required any assistance.

### 2.4. Statistical Analyses

As in the 1st part of the study, descriptive statistics were used to summarize the characteristics of the participants [30]. Continuous variables were presented as means and percentages, while categorical variables were presented as frequencies and percentages.

The original KIDMED scoring system was used, as in the first part of the study [30,31]. The scores were categorized into the following categories: “Very Poor” (≤3), “Needs Improvement” (4–7), and “Optimal” (≥8). A junk food score was calculated based on responses of the four extra dietary questions, which were recategorized into “Low” or “High” consumption, then summed. The final score was recategorized into Low (≤1) and High (≥2) junk food consumption categories.

The normality of all continuous variables was assessed by visually inspecting histograms, and by assessing the results of skewness and kurtosis tests. Non-parametric tests were used when data deviated from normality. The Kruskal–Wallis test was used to assess associations between the categorical KIDMED scores and each of DMFT, decayed teeth (D), and BMI percentiles. Associations between binary junk food consumption questions and DMFT, D, and BMI percentiles were evaluated using the Wilcoxon Rank sum test.

A PA score was calculated by summing the responses of nine physical activity questions. Four of the questions were sports related, and the other five were about PA behaviors inside and out of school. Each activity was recategorized into three levels (low, moderate, high), and the total score was divided by nine to calculate a mean score. The final PA score was then categorized into three groups: low, medium, and high PA level.

A Generalized Linear Model (GLM) with a Gamma family was used to assess the relationship between BMI percentile and the predictor variables: age, sex, DMF, KIDMED category, Junk Food Consumption, and Physical Activity Score. The GLM with a Gamma distribution was used due to the non-normality of the outcome (BMI percentile). Univariate analyses were conducted first, where each independent variable was assessed with the outcome individually, then all independent variables were entered in the model to present the multivariate model.

A *p*-value ≤ 0.05 was considered statistically significant. All statistical analyses were conducted using Stata/BE 18.0 for Mac (StataCorp, College Station, TX, USA).

## 3. Results

Table 1 illustrates the participants’ characteristics. Participants had an equal distribution of males and females and were predominantly Saudi. The majority had a normal BMI range, with a minority classified as overweight, obese, or underweight.

Table 2 presents the descriptive statistics of KIDMED. The majority mean of participants were categorized as having ‘very poor’ or ‘needs improvement’ diets.

Table 3 displays the associations between KIDMED and junk food consumption with decayed teeth (D), DMFT, and BMI percentile. No statistically significant associations between KIDMED or junk food consumption and the outcomes of D, DMFT, or BMI percentile were observed.

As shown in Table 4, most participants had a low level across the different sports assessed. With regard to their activity during school hours, most reported low activity. The overall PA score had a mean of 1.7 ± 0.4, with the scores ranging from 1 to 3.2.

Table 5 presents the associations between PA score and each of KIDMED and DMFT. A significant association was observed between KIDMED and PA. Most of the participants in the low PA category had very poor KIDMED (59.4%), compared to only 0.8% in the optimal KIDMED category. As for DMFT, participants with medium PA levels had a lower DMFT, compared to those with a low PA level.

As illustrated in Table 6, none of the assessed variables showed a statistically significant association with BMI percentile, either in the univariate or the multivariate models.

## 4. Discussion

This study investigated two lifestyle behaviors in senior high school students: diet and PA. The findings revealed that most adolescents (57.7%) had a very poor diet, whereas only 3.3% followed an optimal diet. Most adolescents had low PA levels (81.3%) and only 0.67% engaged in high PA. Nearly 38.7% of the adolescents were overweight/obese and their mean BMI was 24.9 (6.5). Dental caries was also prevalent and the mean DMFT was 4.6 (3.8).

The WHO reported that over 390 million children and adolescents were overweight in 2022; of which, 160 million were obese [33]. In the current study, approximately one-third of the participants were overweight or obese. This is in accordance with previous studies on Saudi youth, except for one study conducted in the Asser region that reported that more than half of the adolescents were overweight/obese [7,34,35]. Conversely, a recent multicenter study found that 20.6% of the study population was overweight or obese, which was lower than that reported in the current study [36].

Obesity in adolescents is multifactorial and has been linked to poor dietary choices, the increased consumption of sweetened beverages, and a sedentary lifestyle [14,16,19]. In the current study, predictors for obesity were assessed. Surprisingly, BMI was not associated with diet quality, junk food consumption, or PA levels. This was unexpected, given the general assumptions and evidence regarding the factors influencing obesity. Several plausible explanations are suggested in understanding these results. First, BMI may not have been the most accurate measure of obesity in this study as it does not account for factors such as body composition and muscle mass. It is also possible that there may have been other confounding factors, such as genetic predisposition, stress, sleep patterns, and hormonal imbalances, that were not investigated or controlled for in the current study, which may have obscured associations that would otherwise be detected. Finally, the cross-sectional assessments of diet and PA might not align with obesity, especially if the effects of these behaviors take longer to manifest in weight changes. Adolescents may have only recently shifted their diets and decreased their PA levels due to the stress of being senior high school students. These suggestions require further investigation. Another finding was that there was no association between BMI and dental caries, which is similar to findings of previous studies on high school students [7,37].

The WHO has identified physical inactivity as the fourth largest risk factor for mortality worldwide [18]. Participants in the current study had a mean PA score of 1.7 (0.4), which is low. In this study, adolescents engaged in running more than other sports; however, 88% still had low running levels. During school recess, only one fourth of the participants were moderately or very physically active. In the first part of the current study, elementary school children reported more activity during school recess, although these levels were also low [30]. Additionally, most participants seemed to engage in minimal PA levels during the week, and only one fourth engaged in moderate and high PA during weekends. The participants in the current study followed recent international trends. A study of over 1.6 million adolescents found that approximately 80% of adolescents globally were insufficiently physically active [38]. Despite inconsistencies in the initiatives studied, a recent review found that adolescents worldwide engage in insufficient levels of PA [21]. These findings are unfortunate, especially in light of growing evidence supporting the health benefits of PA during adolescence. For example, PA in youth has been found to contribute to improved bone density and the preservation of bone loss in adulthood [39]. Moreover, PA has been shown to be useful in improving the mental health and quality of life of adolescents [40]. A systematic review found that PA was inversely associated with body image dissatisfaction, which is common among vulnerable age groups [41].

Participants self-assessed their diets using the KIDMED questionnaire. The KIDMED assesses a person’s adherence to the Mediterranean diet (MD), which is thought to be one of the healthier dietary patterns, relying on a high consumption of plant-based foods, low consumption of red meat, and moderate consumption of fish and dairy products. It also considers the intake of low-glycemic index carbohydrates and unsaturated fats. A high-quality diet, such as the MD, is associated with a more balanced nutrient intake and reduced risk of diet-related non-communicable diseases [42]. Although Saudi Arabia is not a Mediterranean country, KIDMED was used to assess the participants’ diet quality by comparing it to a healthy model, rather than assessing its relevance to the MD per se, as in previous non-Mediterranean studies [43,44,45]. The Saudi diet typically relies on the consumption of rice, red meat, and dairy products. It does not include a variety of fruits or vegetables. Furthermore, the Saudi diet has shifted in recent years toward the Western diet, which involves the consumption of highly processed foods and food high in fats, sugars, and salts [46]. In the current study, more than half the adolescents had a poor diet. Furthermore, many of them were frequently exposed to unhealthy snacks such as chips and sugar-sweetened beverages, including carbonated soft drinks. Similar to our findings, a previous study found that young Saudi adults have a low intake of nuts, fruits, vegetables, and fish, alongside a high consumption of processed foods and sugar-sweetened beverages [47]. A recent study indicated that following the MD can lower the risk of depression, whereas Western dietary patterns may increase the risk and worsen the severity of depression in adolescents [48].

In the current study, no association was detected between dental caries and diet quality, even for sweetened beverages. It is difficult to explain these outcomes, given the abundance of evidence on the direct effect of poor diets, not limited to sugar content, on caries activity [49,50]. There might have been other factors that were not accounted for in the current study, such as oral hygiene habits, fluoride exposure, salivary properties, and access to dental care, which could have attenuated any potential effect of diet quality on oral health within the limitations of this study. Furthermore, investigating the quality and virulence of oral microbiota could have established a more direct link to the development of dental caries, but this was beyond the scope of the present study. Another plausible explanation for the lack of association between diet quality and dental caries is that most participants had a poor diet quality, so the study may not have included sufficient variation to detect an association.

An interesting finding was the positive association between KIDMED scores and PA levels: children who consumed better diets had higher PA levels. This is similar to the findings from recent studies that found that adherence to MD was associated with higher PA, physical fitness, and muscle strength [51,52]. This suggests that a balanced and nutritious diet may be linked to increased motivation or capacity for engaging in PA. Moreover, perhaps the participants who maintained a healthier diet came from households that promoted overall health-conscious behaviors, including regular PA. More interestingly, DMFT differed significantly across physical activity levels, as children with low PA levels had more dental caries than those with higher PA levels. Similar to the current findings, a recent study assessed the influence of regular PA on children’s oral health. The DMFTs were significantly lower in athletic children than in the control group [53]. Another study of Chinese university students found that students with insufficient PA reported more gingival bleeding than more active students [54]. The relationship between PA and oral health may be mediated by other factors. For example, PA influences the composition of saliva, including immunoglobulins and salivary enzymes [55]. PA has also been linked to enhanced immune responses [56]. Another possible explanation goes back to the home environment; it is possible that adolescents who were encouraged to engage in exercise were also encouraged to practice better oral hygiene and had better access to dental care.

It is evident from the current findings that the studied young population led unhealthy lifestyles, by consuming poor diets and not engaging in enough PA. This not only puts them at risk for several health problems, primarily cardiovascular and metabolic disease, but also raises the burden of pediatric obesity [9,57]. It is also evident that obesity and dental caries have multifactorial underpinnings, and that addressing them requires a multifaceted approach. Although some factors, such as genetic predisposition, are difficult to control, others are easier to identify and manage. For instance, staying physically active and adhering to a sustainable diet in adolescence have been shown to decrease the risk of cardiovascular disease in adulthood [58]. Therefore, it is of utmost importance to identify the habits most strongly associated with and predictive of obesity and dental caries, and to focus on modifying them. It is within the scope of the responsibility of healthcare providers who treat adolescents, including pediatric dentists, to provide guidance on the prevention of and reduction of both conditions. Additionally, schools share the responsibility of promoting adolescent health by providing access to healthy food options, limiting sweetened beverages, enhancing access to school facilities, and identifying ways to encourage PA during recesses.

Adolescence is a critical period in lifestyle acquisition [1]. An increased level of maturity offers a greater capacity to understand the importance of health-related habits. With proper education and motivation, health habits that are not adopted in childhood may be gained in adolescence and set the foundation for lifelong healthy behaviors. Research has found that healthy behaviors adopted by youths are likely to be carried into adulthood [59]. Thus, special attention must be paid to adolescents when planning prevention campaigns as this age group is particularly receptive to adopting habits that can influence their long-term health and well-being.

This study has certain limitations that must be addressed. First, its cross-sectional nature may have prevented the establishment of causal relationships from the observed associations. Second, the sample size may have been too small to have the statistical power to show significant relationships. Second, the use of self-reported physical activity could lead to discrepancies between actual and perceived reported PA. In our study, reported PA was low, which makes it less likely that the low PA was under reported. Finally, the lack of a standardized healthy Saudi dietary reference has led researchers to assess diet quality using the MD as a benchmark. Nevertheless, this study had notable strengths. First, a validated questionnaire was administered. Second, the methodology was thorough and reproducible. This study opens new avenues for future research by highlighting poor diets and sedentary lifestyles among Saudi youth and exploring associations that have rarely been addressed in the literature. Additionally, these findings provide a base for further investigation to gain a deeper understanding of the complex etiology of obesity and dental caries in adolescents.

## 5. Conclusions

The adolescents in this study self-reported poor-quality diets based on KIDMED and minimal PA level based on PAQ-A. No associations could be found between PA and BMI. Also, no associations could be found between diet and either BMI or dental caries. However, PA was positively correlated with diet quality and inversely correlated with the incidence of dental caries. These results highlight the need for multilevel interventions to address unhealthy lifestyle habits, ranging from individual behavior modification to broader community-based initiatives. Such efforts may include nutrition education campaigns, school and community programs promoting physical activity, enhancing school meal quality, and forming partnerships with healthcare professionals. Further longitudinal research on a large scale is encouraged to gain a better understanding of the relationship between lifestyle habits and obesity and dental caries.

## Figures and Tables

**Table 1:** Characteristics of the children (N = 300), using the template of the 1st study [30].

Characteristic	N (%)
**Age**, mean (SD)	18.6 (0.5)
**Nationality**	
Saudi	266 (88.7)
Non-Saudi	34 (11.3)
**Sex**	
Male	150 (50.0)
Female	150 (50.0)
**Weight**, mean (SD)	67.1 (19.7)
**Height**, mean (SD)	164.2 (9.1)
**BMI**, mean (SD)	24.9 (6.5)
**BMI percentile**, mean (SD)	59.4 (32.4)
**BMI percentile**, categorical	
Underweight	11 (3.7)
Normal	173 (57.6)
Overweight/obese	116 (38.7)
**Decayed**, mean (SD)	3.2 (3.5)
**DMFT**, mean (SD)	4.6 (3.8)

**Table 2:** The descriptive statistics of KIDMED score among the participants, using the template of the 1st study [30].

KIDMED Score Statistics	Value
Mean (SD)	3.0 (2.5)
Median (IQR)	3 (1, 5)
Range	−4, 10
**Categories**	
Very Poor (≤3)	173 (57.7)
Needs Improvement (4–7)	117 (39.0)
Optimal (≥8)	10 (3.3)

**Table 3:** Distribution of KIDMED scores and associations with D, DMFT, and BMI percentile, using the template of the 1st study [30].

Item	D, Mean (SD)	*p*-Value	DMFT, Mean (SD)	*p*-Value	BMI Percentile	*p*-Value
**KIDMED score**						
Very poor	3.2 (3.5)	0.766 #	4.7 (3.8)	0.766 #	58.7 (33.9)	0.667 #
Needs improvement	3.2 (3.6)		4.4 (3.9)		60.0 (30.0)	
Optimal	2 (2.2)		4.4 (3.0)		65.1 (35.7)	
**Daily chips consumption**						
No	3.3 (2.8)	0.354 ^	5.1 (3.8)	0.451 ^	55.0 (32.8)	0.390 ^
Yes	3.2 (3.6)		4.6 (3.8)		59.9 (32.4)	
**Daily bottled juice consumption**						
No	2.8 (3.1)	0.550 ^	4.2 (3.4)	0.413 ^	63.2 (31.4)	0.210 ^
Yes	3.3 (3.7)		4.7 (3.9)		58.0 (32.8)	
**Daily flavored milk consumption**						
No	2.9 (3.3)	0.417 ^	4.3 (3.6)	0.357 ^	61.2 (31.6)	0.659 ^
Yes	3.3 (3.6)		4.7 (3.9)		58.9 (32.7)	
**Daily soft drinks consumption**						
No	3.1 (3.5)	0.979 ^	4.9 (3.7)	0.953 ^	63.7 (30.0)	0.387 ^
Yes	3.2 (3.5)		4.6 (3.8)		58.4 (32.9)	

# Kruskal–Wallis, ^ Wilcoxon Rank sum test.

**Table 4:** Distribution of sports/PA among the participants, using the template of the 1st study [30].

Variable	N (%)
**Have you done any of the following activities in the past 7 days (last week)? If yes, how many times?**	
**Swimming**	
Low activity	296 (98.7)
Moderate activity	4 (1.3)
High activity	0 (0.0)
**Football**	
Low activity	294 (98.0)
Moderate activity	5 (1.7)
High activity	1 (0.3)
**Basketball**	
Low activity	294 (98.0)
Moderate activity	3 (1.0)
High activity	3 (1.0)
**Running**	
Low activity	264 (88.0)
Moderate activity	21 (7.0)
High activity	15 (5.0)
**In the last 7 days, what did you do most of the time at recess?**	
Low activity	220 (73.3)
Moderate activity	67 (22.3)
High activity	13 (4.3)
**In the last 7 days, what did you normally do at lunch (besides eating lunch)?**	
Low activity	257 (85.7)
Moderate activity	40 (13.3)
High activity	3 (1.0)
**In the last 7 days, on how many days did you do sports, dance, or play games in which you were very active after school?**	
Low activity	262 (87.3)
Moderate activity	26 (8.7)
High activity	12 (4.0)
**On the last weekend, how many times did you do sports, dance, or play games in which you were very active?**	
Low activity	227 (75.7)
Moderate activity	67 (22.3)
High activity	6 (2.0)
**How do you describe yourself in terms of PA last week?**	
Low activity	107 (35.7)
Moderate activity	182 (60.7)
High activity	11 (3.7)
**Physical activity score**	
Mean (SD)	1.7 (0.4)
Range	1, 3.2
Low PA (mean of 1–2)	244 (81.3)
Medium PA (mean of 3)	54 (18.0)
High PA (mean of 4–5)	2 (0.67)

**Table 5:** Distribution of KIDMED and DMFT across PA categories.

	KIDMED					DMFT			Difference Between the Groups
PA	Very Poor (N, %)	Needs Improvement (N, %)	Optimal (N, %)	Total (N, %)	*p*-Value *	Mean (SD)	Median (IQR)	*p*-Value ^	*p*-Value #
**Low**	145 (59.43)	97 (39.75)	2 (0.82)	244 (100)	<0.001	4.86 (3.84)	4 (7)	0.0295	Low vs. Medium: 0.01 Low vs. High: 0.630 Medium vs. High: 0.309
**Medium**	28 (51.85)	20 (37.04)	6 (11.11)	54 (100)		3.43 (3.43)	3 (6)		
**High**	0 (0.00)	0 (0.00)	2 (100)	2 (100)		5.50 (0.71)	5.5 (1)		
**Total**	173 (57.67)	117 (39.00)	10 (3.33)	300 (100)		---	---		

* Fisher’s exact test, ^ Kruskal–Wallis, # Wilcoxon Rank sum test.

**Table 6:** Predictors of BMI percentile, using the template of the 1st study [30].

Variable (Categories)	Univariate Analysis (Coefficient (95% CI))	Multivariate Analysis (Coefficient (95% CI))
**Age**	−0.05 (−0.18, 0.08)	−0.06 (−0.19, 0.08)
**Sex**		
Male	Reference	Reference
Female	−0.02 (−0.15, 0.10)	−0.04 (−0.17, 0.09)
**DMF**	−0.02 (−0.02, 0.01)	−0.00 (−0.02, 0.01)
**KIDMED**		
Very Poor	Reference	Reference
Needs Improvement	0.02 (−0.11, 0.15)	0.03 (−0.10, 0.16)
Optimal	0.10 (−0.25, 0.45)	0.04 (−0.35, 0.44)
**Junk Food**		
Low Consumption	Reference	Reference
High Consumption	0.10 (−0.05, 0.24)	0.11 (−0.04, 0.26)
**PA Score**	0.10 (−0.07, 0.27)	0.08 (−0.11, 0.27)

Note: Generalized Linear Model (GLM) was used.

## Data Availability

All data generated for this study are available from the corresponding author upon reasonable request. The data are not publicly available to protect data privacy and restrict unauthorized use.

## List of Abbreviations

**BMI:** Body Mass Index; **CI:** Confidence Interval; **DMFT:** decayed, missed, filled teeth score; **DS:** Deanship of Scientific Research; **KAUFD:** Faculty of Dentistry at King Abdulaziz University; **KIDMED:** The Mediterranean Diet Quality Index; **MD:** Mediterranean Diet; **PA:** Physical Activity; **PAQ-C:** Physical Activity Questionnaire for Older Children; **SD:** Standard Deviation; **WHO:** World Health Organization.
